# Splenic Infarct as the Presenting Manifestation of Essential Thrombocythemia

**DOI:** 10.31486/toj.21.0073

**Published:** 2022

**Authors:** Shalaka Khade, Sudeep Khera, Vaibhav Kumar Varshney, Deepak Kumar Sharma, Raghav Nayar, Abhishek Purohit

**Affiliations:** ^1^ Department of Pathology, All India Institute of Medical Sciences, Jodhpur, Rajasthan, India.; ^2^ Department of Surgical Gastroenterology, All India Institute of Medical Sciences, Jodhpur, Rajasthan, India; ^3^ Department of Medicine, All India Institute of Medical Sciences, Jodhpur, Rajasthan, India

**Keywords:** *Splenic infarction*, *thrombocythemia–essential*, *thrombosis*

## Abstract

**Background:** Essential thrombocythemia is a chronic myeloproliferative neoplasm characterized by thrombotic and hemorrhagic complications. Essential thrombocythemia can be considered a risk factor for thrombotic events.

**Case Report:** A 34-year-old female presented with sudden onset of abdominal pain from splenic infarction for which she underwent splenectomy. Bone marrow examination performed because of increasing thrombocytosis led to a diagnosis of essential thrombocythemia. Postoperatively, she was maintained on low-dose aspirin and doing well at follow-up.

**Conclusion:** Our patient had an undiagnosed case of essential thrombocythemia and presented with symptoms related to splenic infarction. To the best of our knowledge, few cases of splenic infarction consequent to essential thrombocythemia have been reported.

## INTRODUCTION

Essential thrombocythemia, a chronic myeloproliferative neoplasm, is characterized by persistent thrombocytosis. While essential thrombocythemia is frequently diagnosed incidentally,^1^ some cases present with thrombotic or hemorrhagic complications.^2^ The increased risk of hemorrhagic complications can be associated with extreme thrombocytosis, but the cause of thrombotic complications is not well understood. Thrombotic complications vary from mild disturbances to severe events.^3^ Although thrombotic complications are known to occur in essential thrombocythemia, few cases have been reported with splenic infarction as the presenting feature. Splenic infarction is an uncommon diagnosis that can be caused by trauma, embolization, infection, hematologic conditions, or malignancy.^4^ Splenic infarction occurs as a result of occlusion of the splenic artery or one of its branches by an embolus or in situ thrombosis. The spleen has a rich vascular supply and receives approximately 5% of cardiac output, making it susceptible to emboli formation.^5^ Also, the spleen is commonly affected by hematologic malignancies, which can increase the risk of thrombosis.^6,7^ We describe a patient with essential thrombocythemia who presented with symptoms arising from splenic infarction.

## CASE REPORT

A 34-year-old female presented with severe pricking pain in the left upper abdomen that began 6 days prior, was not relieved with oral analgesics, and was associated with multiple episodes of nonprojectile vomiting. The patient had no history of hypertension or cardiovascular disease and no relevant family history. The primary workup, a blood transfusion, and initial imaging had been done elsewhere before the patient was referred to our institute for further management. Imaging indicated an evolving pseudopancreatic cyst with an ischemic insult of the spleen. On examination in our emergency department, the patient had stable vital signs and tender, moderate splenomegaly. Contrast-enhanced computed tomography repeated at our institution revealed splenic infarction with contained splenic rupture, mild ascites, and left-sided pleural effusion.

Hemoglobin was 7.6 g/dL (reference range, 12-15 g/dL), total leukocyte count was 27 × 10^9^/L (reference range, 4-11 × 10^9^/L), and platelet count was 970 × 10^9^/L (reference range, 150-450 × 10^9^/L). Serum iron studies and serum ferritin revealed evidence of iron deficiency: total iron-binding capacity was 547 μg/dL (reference range, 240-450 μg/dL) and iron was 22 μg/dL (reference range, 60-170 μg/dL).

The patient's case was reviewed in the hematology clinic. The patient was administered iron sucrose 5 mL of 5 mg/mL solution diluted in 100 mL normal saline for 3 consecutive days as per the advice of the hematologist. The patient was initiated on low-dose aspirin because of thrombocytosis. She was managed conservatively for 6 days, during which complete blood counts, iron studies, and inflammatory markers (high sensitivity C-reactive protein and erythrocyte sedimentation rate) were evaluated. She was started on broad-spectrum antibiotics (meropenem injection 1 g intravenously 3 times daily) and continued on the same iron therapy as mentioned above because of leukocytosis and thrombocytosis. Full procoagulant workup (serum homocysteine, fibrinogen, antinuclear antibodies, protein C, protein S, antithrombin III) was negative.

After ruling out all possible causes of secondary thrombosis and given the nonresolution of the patient's symptoms, the decision was made to operate. Our routine protocol is to provide vaccination 14 days before elective splenectomy against *Haemophilus influenza, pneumococcus*, and *meningococcus*. Because the patient was taken for surgery on a semi-emergency basis, she could not be vaccinated preoperatively, so vaccination was planned for the postoperative period. She underwent exploratory laparotomy with splenectomy and drainage of the lesser sac collection (approximately 100 mL), which was sterile on culture. Densely thickened and inflamed omentum was wrapped around a devascularized and ruptured spleen.

Histopathologic examination of the splenectomy specimen revealed predominantly infarcted areas with only focally preserved splenic parenchyma, indicating massive splenic infarction (Figure).

**Figure.**
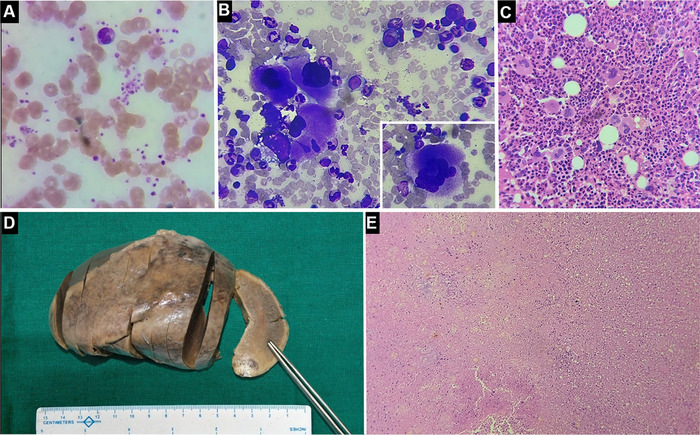
(A) Peripheral blood smear (Leishman stain, magnification ×100) shows increased platelets and platelet clumps. (B) Bone marrow aspirate (Giemsa stain, magnification ×40) shows cellular marrow with megakaryocytes cluster (inset: hyperlobated megakaryocyte). (C) Bone marrow biopsy shows increased number of megakaryocytes with hyperlobated nuclei (hematoxylin and eosin stain [H&E], magnification ×40). (D) Photograph of resected spleen with pale cut surface. (E) Photomicrograph of spleen shows infarcted areas (H&E, magnification ×40).

Postoperatively, the patient's platelet count progressively increased (reaching 1,638 × 10^9^/L during the next 8 days), presumably as an acute response to splenectomy. Other reactive causes such as connective tissue disorders, hemolytic anemia, medications, and inflammatory states were ruled out based on history and ancillary laboratory investigations. The ongoing increase in platelet count led us to perform a bone marrow examination on the eighth day of admission that revealed a cellular bone marrow with marked megakaryocytic prominence. Megakaryocytes displayed large to giant forms with abundant mature cytoplasm and conspicuously hypersegmented staghorn–like nuclei. These findings were consistent with essential thrombocythemia.

After a hospital stay of 10 days, the patient was discharged on oral cefuroxime 500 mg twice daily, aceclofenac 100 mg twice daily, and pantoprazole 40 mg once daily for 5 days. She returned for follow-up after 7 days for suture removal and laboratory investigations.

Molecular studies were performed to complete the workup for essential thrombocythemia. The test was negative for the BCR-ABL1 mutation, but the JAK2 V617F mutation was detected, meeting the criteria of essential thrombocythemia.

The patient was vaccinated 1 month postoperatively and maintained on oral aspirin 75 mg once daily. She was followed up every 3 months during the first year and every 6 months during subsequent years. Her platelet count stabilized to 380 × 10^9^/L after 1 month, so she did not require chemotherapy. She continues to do well after 18 months of follow-up with no evidence of infection.

## DISCUSSION

This report demonstrates a previously undiagnosed case of essential thrombocythemia in a patient who presented with symptoms related to splenic infarction. Our patient presented with sudden onset of acute abdominal pain that could be a consequence of thrombotic episode leading to massive splenic infarction. To the best of our knowledge according to a literature search, few cases of splenic infarction as a result of essential thrombocythemia have been reported (Table).^3,7-14^ Other rare presentations of essential thrombocythemia that have been reported are aortic thrombosis, cerebral infarct, and one case of multiple venous and arterial thromboses of the gallbladder causing acute cholecystitis.^3,11-14^ One patient demonstrated major vascular complications of essential thrombocythemia, including multiple splenic infarctions, and in later years, the patient developed cerebral infarction and myocardial infarction.^14^

**Table.** Cases of Essential Thrombocythemia With Splenic Infarction

Study	Age/Sex	Symptoms	Platelet count, × 10^9^/L
Yamaguchi et al, 1985^8^	64/M	Fever, left abdominal pain	584
Aoyama et al, 1988^9^	48/M	Left back pain	927
Tohyama and Haruhiko, 1996^10^	68/F	Epigastric pain	856
Oki et al, 2008^11^	65/F	Lower abdominal pain, vomiting, diarrhea	1,665
Bachmeyer and Elalamy, 2011^12^	64/F	Left hypochondrial pain radiating to back	540
Picón-Coronel et al, 2011^13^	22/M	Right sided abdominal pain (cholecystitis)	2,060
Kim et al, 2012^14^	46/M	Splenic infarctions, myocardial infarction, cerebral infarction	>600
Yuan et al, 2018^3^	63/F	Numbness and weakness in right extremities	448
Yoshida et al, 2019^7^	31/M	Left hypochondrial pain	436
Present case, 2022	34/F	Left upper abdominal pain, vomiting	970

Various retrospective studies demonstrated that the frequency of thrombotic complications ranges from 31% to 83%; however, the occurrence of deep vein thrombosis including splenic, portal, and hepatic vein thrombosis is very low, around 4%.^2^ Although risk factors such as age, duration of thrombocytosis, and history of thrombosis have been postulated in some reports, no clear correlation has been established.^11,15^ Yoshida et al postulated increased platelet count and dysfunctional cell surface causing increased adhesion of platelets as the mechanism of thrombosis in essential thrombocythemia.^7^ Essential thrombocythemia can be considered a risk factor for thrombotic events. Early diagnosis and management of essential thrombocythemia can help prevent major thrombotic events such as massive splenic infarction, cerebral infarction, and aortic infarction.^3^ Establishing some working criteria for early identification of individuals who are at increased risk is important. Risk stratification by considering history of thrombosis, JAK2 and MPL mutations, and age can be beneficial, and treatment can be planned accordingly.^16^ Treatment for essential thrombocytosis includes cytoreductive therapy with hydroxyurea. Anagrelide is an alternative second-line therapy in patients who do not tolerate hydroxyurea or do not respond to aspirin.^17^

## CONCLUSION

Although hemorrhagic and thrombotic complications are known to occur with essential thrombocythemia, splenic infarction is rare. Early recognition with risk stratification and management of essential thrombocythemia could help avert such thrombotic manifestations.

## Notes

*This article meets the Accreditation Council for Graduate Medical Education and the American Board of Medical Specialties Maintenance of Certification competencies for Patient Care and Medical Knowledge.*
